# MicroRNA-199b Deregulation Shows Oncogenic Properties and Promising Clinical Value as Circulating Marker in Locally Advanced Rectal Cancer Patients

**DOI:** 10.3390/ijms23042203

**Published:** 2022-02-17

**Authors:** Andrea Santos, Ion Cristóbal, Jaime Rubio, Cristina Caramés, Melani Luque, Marta Sanz-Alvarez, Miriam Morales-Gallego, Juan Madoz-Gúrpide, Federico Rojo, Jesús García-Foncillas

**Affiliations:** 1 Cancer Unit for Research on Novel Therapeutic Targets, Oncohealth Institute, IIS-Fundación Jiménez Díaz-UAM, 28040 Madrid, Spain; andrea.santos@quironsalud.es (A.S.); jaime.rubiop@quironsalud.es (J.R.); ccarames@fjd.es (C.C.); 2 Translational Oncology Division, Oncohealth Institute, IIS-Fundación Jiménez Díaz-UAM, 28040 Madrid, Spain; 3 Medical Oncology Department, University Hospital “Fundación Jiménez Díaz”, UAM, 28040 Madrid, Spain; 4 Pathology Department, IIS-Fundación Jiménez Díaz-UAM, 28040 Madrid, Spain; melani.luque@quironsalud.es (M.L.); marta.sanza@quironsalud.es (M.S.-A.); miriam.moralesg@quironsalud.es (M.M.-G.); jmadoz@fjd.es (J.M.-G.); frojo@fjd.es (F.R.)

**Keywords:** MiR-199b, tumor suppressor, prognosis, LARC

## Abstract

The identification of robust prognostic markers still represents a need in locally advanced rectal cancer (LARC). MicroRNAs (miRs) have progressively emerged as promising circulating markers, overcoming some limitations that traditional biopsy comprises. Tissue miR-199b deregulation has been reported to predict outcome and response to neoadjuvant chemoradiotherapy (nCRT) in LARC, and was also found to be associated with disease progression in colorectal cancer. However, its biological and clinical relevance remains to be fully clarified. Thus, we observed here that miR-199b regulates cell migration, aggressiveness, and cell growth, and inhibits colonosphere formation and induces caspase-dependent apoptosis. Moreover, miR-199b expression was quantified by real-time PCR in plasma samples from LARC patients and its downregulation was observed in 22.7% of cases. This alteration was found to be associated with higher tumor size (*p* = 0.002) and pathological stage (*p* = 0.020) after nCRT. Notably, we observed substantially lower global miR-199b expression associated with patient downstaging (*p* = 0.009), as well as in non-responders compared to those cases who responded to nCRT in both pre- (*p* = 0.003) and post-treatment samples (*p* = 0.038). In concordance, we found that miR-199b served as a predictor marker of response to neoadjuvant therapy in our cohort (*p* = 0.011). Altogether, our findings here demonstrate the functional relevance of miR-199b in this disease and its potential value as a novel circulating marker in LARC.

## 1. Introduction

Colorectal cancer (CRC) is already the third leading cause of cancer death in the world, with rectal cancer (RC) representing almost 30% of total CRC cases, and its incidence is steadily rising in developing countries and in younger patients [1]. According to GLOBOCAN data, CRC is also the third most commonly diagnosed form of cancer globally, comprising 10% of all cancer diagnoses [2]. In Spain, around 43,600 new cases of CRC have been diagnosed in 2021, from which more than 14,200 were RC patients [3]. Locally advanced rectal cancer (LARC), defined as clinical stage II (T3-4, lymph node negative) or stage III (lymph node positive) disease has experienced a great paradigm shift in management over the past few decades given the anatomical constrictions of the pelvis and the risk for local recurrence and distant metastasis. Despite advances in preoperative treatment and surgical techniques, response to neoadjuvant chemoradiotherapy (nCRT) varies among patients and, while nearly 40% of LARC patients achieve partial responses, about 20% have a pathological complete response (pCR). Thus, health-related quality of life and survival still remain suboptimal in patients with this disease [4,5]. Moreover, given the achievement of pCR in a wide range of patients undergoing nCRT and the major effects and complications of total mesorectal excision (TME) surgery, there is a growing interest in the identification of patients that could benefit from a watch and wait (W&W) clinical approach. This novel strategy could avoid surgery in around 25% of cases and supports organ preservation, avoiding unnecessary postoperative morbidity with good long-term oncological outcomes and improving quality of life in highly selected patients [6,7]. Thus, the RAPIDO and PRODIGE 23 phase III randomized clinical trials have recently shown that the addition of neoadjuvant chemotherapy to a standard short- or long-course radiation significantly decreases the risk of metastatic progression and associates with a better disease-free survival in LARC patients [8,9].

In the last decades, advances in medical oncology have improved the treatment of patients and their outcomes, but the current use of traditional biopsies based on obtaining tumor tissue has several limitations in the developing era of precision medicine, mainly due to the progression of cancer and the onset of therapy resistance [5]. In this regard, research is constantly moving forward to find more accurate and personalized biomarkers [10]. In fact, there is a critical need to identify novel biomarkers with high specificity and sensitivity in LARC patients [11,12]. Liquid biopsy represents a promising tool for biomarker detection due to its potential to overcome many of the limitations of classical biopsy. The obtention of liquid biopsies is minimally invasive and can be repeated several times. Moreover, it is a cost-effective method that can be used to screen and monitor both disease evolution and treatment response as well as to identify cellular subclones involved in relapse, metastasis or treatment resistance [13,14]. Liquid biopsy refers to the isolation of cancer-derived components, such as circulating tumor cells (CTC), circulating tumor DNA (ctDNA), microRNAs (miRs), long non-coding RNAs (lncRNAs) and proteins, from peripheral blood or other body fluids, and their genomic or proteomic assessment [15]. In this context, miRs appear to be good candidates as liquid biopsy-based cancer biomarkers. They are gaining more attention than other potential biomarkers detectable in liquid biopsies, and seem to be potential candidates in predicting LARC and CRC prognosis and therapy response, since they are easily detectable, highly stable in biological fluids, and show higher sensitivity and specificity compared to other circulating tumor components [14,16,17].

MiRs are highly conserved endogenous non-coding and single-stranded RNAs of 18–25 nucleotides in length. Generally, miRs negatively regulate gene expression via binding to the 3′-untranslated region (3′-UTR) of their target mRNAs that result in transcriptional repression with or without mRNA degradation [17,18,19,20]. Previous studies have indicated that altered expression levels of circulating miRs are related to cellular transformation and carcinogenesis progression in different tumor types [21,22,23,24,25,26,27,28,29]. MiRs have been implicated in development and progression of CRC by functioning as oncogenes or tumor suppressors [30]. MiR-199b downregulation has been associated with tumorigenesis and metastasis in various human cancers through the alteration of different signaling pathways. In fact, miR-199b overexpression has been reported to decrease proliferation, migration and invasion in hepatocellular carcinoma cells by directly binding and negatively regulating JAG1, thereby influencing Notch signaling [31,32]. The Notch signaling pathway has been described to be indirectly regulated in medulloblastoma by miR-199b. This miR negatively correlates with HES1, a key Notch effector, impairing CD133+ stem cell-like subpopulation of cancer cells, demonstrating the key role of miR-199b in directly targeting CD133 expression [33,34]. Moreover, this miR has a significant tumor suppressive function in the invasion and metastasis of prostate and triple negative breast cancers [35,36] regulating epithelial to mesenchymal transition (EMT) through the inhibition of the DDR1-ERK signaling axis. It has been described that miR-199b is repressed in breast cancer cells, and its overexpression reduces tumor growth and angiogenesis by directly targeting ALK1 [37]. All of these targets have also been described to be deregulated in CRC, which would confirm the possible contributing role of miR-199b in tumor progression and metastasis through the regulation of multiple signaling pathways. CircNSD2 was found to target miR-199b in CRC cells leading to DDR1/JAG1 activation and promoting the development of metastatic disease [38]. Our group has previously reported that miR-199b could be involved in regulating tumor stemness through CD133 modulation, since a negative correlation between this miR and CD133 in CRC cell lines has been shown and further confirmed in metastatic CRC patients [39]. Moreover, it has been described that the deregulation of the ALK1/TGF-β signaling pathway enhanced both EMT of tumor cells and self-renewal ability of cancer stem cells in CRC, thereby contributing to disease progression, suggesting a role of miR-199b in this issue [40,41], as it occurs in other tumor types described above. Notably, miR-199b downregulation has been described to be associated with increased cell invasion and migration in CRC, promoting cancer progression and metastasis by SIRT1/CREB/KISS1 signaling pathway regulation, SIRT1 being a direct target of this miR [42]. The contribution of the miR-199b target SET to these functional effects with a particular interest in LARC should also be considered. Thus, patients with LARC are treated with a 5-fluorouracil (5-FU) based preoperative CRT, and it has been reported that SET determines CRC cell sensitivity to this chemotherapeutic agent [43,44].

In summary, miR-199b has emerged as a promising clinical biomarker for CRC and LARC patients [45,46], but some aspects regarding miR-199b-induced effects at the functional level in this disease still remain to be fully clarified, as well as its potential clinical usefulness not only as a tissue tumor marker but also as a circulating marker.

In this report, we aimed to further explore the biological role of miR-199b expression in disease progression and analyze its potential value as a circulating marker in LARC. Thus, we observed that miR-199b regulates cell migration and aggressiveness, inhibits colonosphere formation and induces caspase-dependent apoptosis in CRC cells. Moreover, we studied miR-199b expression in plasma samples of LARC patients, observing that its downregulation is a common event strongly associated with relapse and lack of response to nCRT. Clinically, our results here are concordant with our previous findings in tumor biopsies and provide a preliminary basis for the use of miR-199b as a circulating marker in LARC patients.

## 2. Results

### 2.1. MiR-199b Regulates Cell Migration and Agressiveness

**Figure 1:**
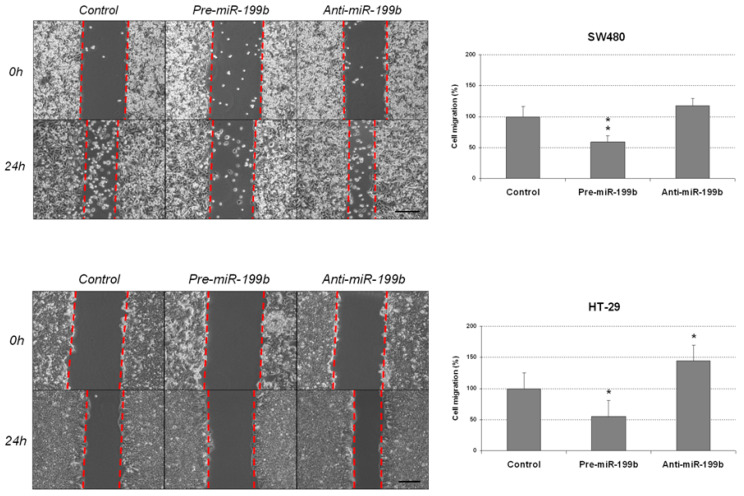
MiR-199b modulates cell migration in CRC cells. Wound-healing assay showing migration of SW480 and HT-29 cell lines after transfection with pre-miR-199b and anti-miR-199b. Dashed lines represent the migration border; * *p* < 0.05; ** *p* < 0.01. Scale bar: 200 µm.

We aimed to investigate the functional relevance of miR-199b deregulation as an alteration that could be contributing to disease progression. We started analyzing potential changes in cell migration of the CRC cell lines SW480 and HT-29 after an ectopic modulation of this miR. Quantification of miR-199b was performed to confirm the efficacy of the transfections (Table S1). We performed wound-healing assays and observed that miR-199b overexpression substantially reduced migration in SW480 cells, whereas the opposite effect was found in the same cell line after miR-199b silencing. However, results did not achieve statistical significance in SW480 cells transfected with anti-miR-199b. These observations were confirmed in the HT-29 cell line, which showed similar regulation of its migration ability when miR-199b was overexpressed or silenced, respectively, and differences were statistically significant in both cases with this cell line (Figure 1).

Given that SET has been reported in previous works from our group as a key direct target of miR-199b, we also analyzed the role of the miR-199b/SET axis in cell migration. We first confirmed the previously published role of miR-199b as a direct negative SET regulator (Figure S1). Next, we performed transwell migration assays showing that SET overexpression totally restored the miR-199b-derived inhibition of cell migration in both SW480 and HT-29 cells (Figure S2).

**Figure 2:**
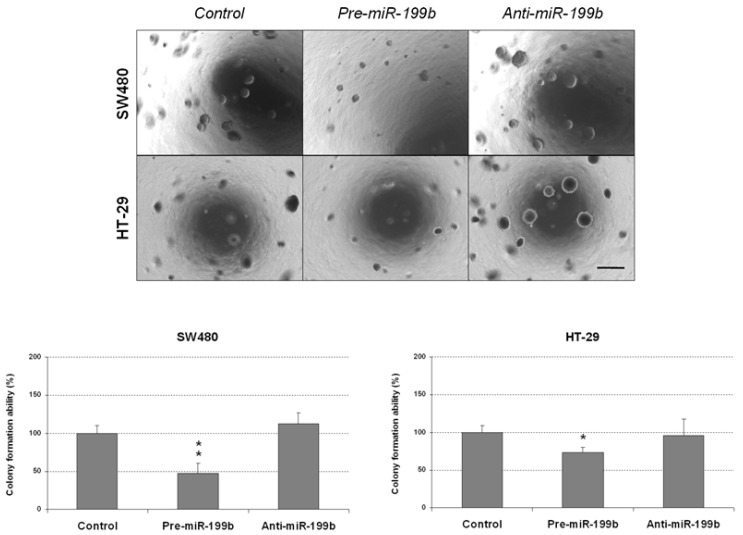
MiR-199b deregulation affects the colony-forming ability of CRC cells. Colony-forming assays showing the effect on the anchorage-independent cell growth of SW480 and HT-29 cell lines after ectopic miR-199b silencing or overexpression; * *p* < 0.05; ** *p* < 0.01. Scale bar: 200 µm.

To confirm the antitumor effects of miR-199b, we next performed colony-formation assays in soft agar to analyze whether this miR could also affect the aggressiveness of CRC cells by altering its anchorage-independent growth ability. We observed that colony formation was markedly inhibited in both SW480 and HT-29 cells ectopically overexpressing miR-199b. Of note, we did not find changes in these cell lines after miR-199b silencing compared with the negative controls (Figure 2). As expected, we found that the ectopic expression of SET was able to totally restore the miR-199b-induced effects in both the SW480 and HT-29 cell lines (Figure S3A). We also observed marked differences in the size of the colonies formed with the SW480 cell line (Figure S3B). Based on these findings, we also evaluated the role of this signaling axis in CRC cell growth. In concordance with soft agar assays, we observed that ectopic expression of SET significantly restored the anti-proliferative effects of miR-199b in both SW480 and HT-29 cells (Figure S4). Altogether, our observations indicate that miR-199b plays a role in disease progression and aggressiveness by regulating cell migration and the colony-forming ability of CRC cells.

### 2.2. MiR-199b Induces Apoptosis and Inhibts Colonosphere Formation

**Figure 3:**
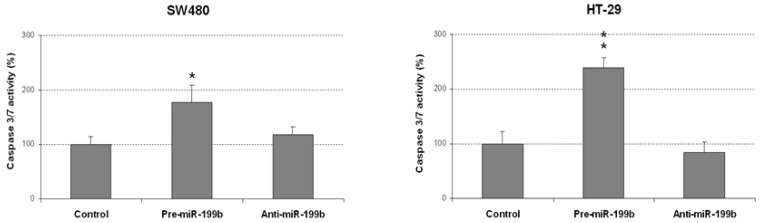
MiR-199b induces apoptosis in CRC cells. Caspase-3/7 activity assay showing levels of apoptotic SW480 and HT-29 cells after transfection with pre-miR-199b and anti-miR-199b; * *p* < 0.05; ** *p* < 0.01.

The markedly reduced size of the colonies obtained in colony-forming assays prompted us to analyze whether miR-199b could be affecting apoptosis in CRC cells. Thus, we observed that miR-199b overexpression led to enhanced caspase-dependent apoptosis in both SW480 and HT-29 cells (Figure 3). However, we did not find differences in the apoptotic levels of CRC cell lines after miR-199b silencing compared to negative controls, which was in concordance with the results obtained in soft agar experiments (Figure 2).

**Figure 4:**
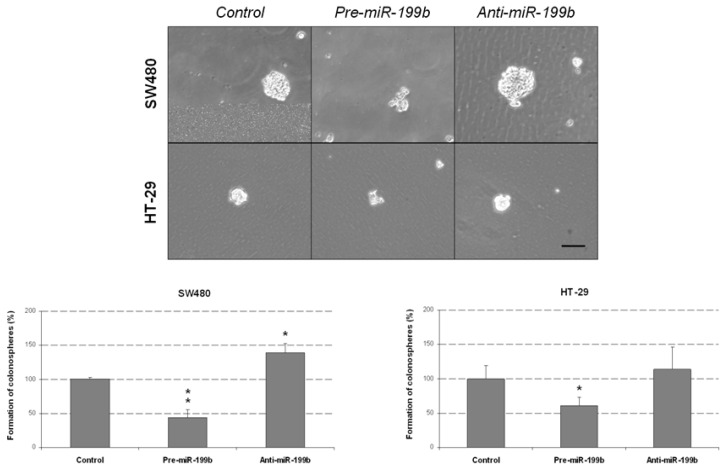
MiR-199b negatively regulates colonosphere formation from CRC cells. Optical microscope images showing the colonospheres formed in the different conditions. The graphs show the number of SW480 and HT-29-derived colonospheres obtained after transfection with pre-miR-199b and anti-miR-199b; * *p* < 0.05; ** *p* < 0.01. Scale bar: 200 µm.

We further explored the biologic effects of miR-199b deregulation in CRC by analyzing the colonosphere formation ability of SW480 and HT-29 cells. Notably, miR-199b overexpression led to significant decreased colonosphere formation ability in both number of colonospheres formed (Figure 4) and cells per colonosphere (Figure S5), suggesting that miR-199b would be playing an important role in colonosphere formation and self-renewal of CRC cells. Conversely, colonosphere formation was enhanced by the transfection with anti-miR-199b in both cell lines, but significance was only achieved in SW480 cells.

### 2.3. Analysis of miR-199b Expression Levels as Circulating Markers in Larc Patients and Its Association with Molecular and Clinical Parameters

To evaluate the potential usefulness of miR-199b as a circulating marker in LARC, we obtained liquid biopsies from a series of 22 patients that were diagnosed by LARC, underwent 5-FU-based nCRT in Fundación Jiménez Díaz Hospital and had clinical follow-up data available. A summary of the molecular and clinical characteristics of the cohort studied is shown in Table S2, and the same information of each case included in the study is provided in Table S3. We observed miR-199b downregulation in 22.7% of cases (5 out of 22). Notably, we found a significant correlation between miR-199b downregulation and higher tumor size after nCRT (*p* = 0.002), as well as higher pathological stage (*p* = 0.020). Interestingly, patients with low miR-199b levels also showed correlation with lymph node positivity after nCRT, but statistical significance was not achieved in this case (*p* = 0.091). The association between miR-199b expression and clinical and molecular characteristics is included in Table 1.

### 2.4. Circulating miR-199b Expression Levels Predict Pathological Response to nCRT and Recurrence in LARC Patients

**Figure 5:**
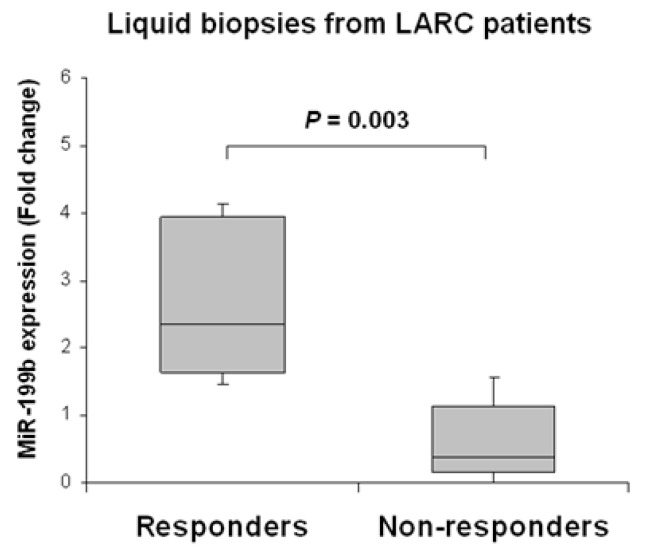
MiR-199b downregulation associates with lack of response to nCRT in pre-treatment samples of LARC patients. The box-plot shows miR-199b expression levels in LARC patients stratified by response or non-response to nCRT treatment. The responder group corresponds to cases with moderate or complete response (RYAN 0 and 1), including cases following the W&W protocol. The non-responder group includes those with minimal or none complete response (RYAN 2 and 3).

We next evaluated the clinical impact of miR-199b as a predictor of response to preoperative CRT in LARC. Our patient cohort was stratified into responders and non-responders to neoadjuvant therapy. As a preliminary analysis we compared global miR-199b expression between both subgroups, observing that the subgroup of non-responders had substantially lower miR-199b levels than responder patients (*p* = 0.003) (Figure 5).

In concordance with these observations, we found that miR-199b downregulation served as a circulating marker predictive of lack of response to nCRT in our patient cohort (*p* = 0.011) (Table 2).

To further evaluate the clinical relevance of miR-199b as a novel circulating marker in LARC patients, we also studied its prognostic value to predict both patient recurrence and downstaging. MiR-199b was found downregulated in two out the three cases with a reported recurrence in our cohort, and the association was very close but failed to achieve statistical significance in this case (*p* = 0.051) (Table S4). However, we observed that the subgroup of cases with low miR-199b levels was significantly associated with a lack of downstaging when we compared the clinical stage before and after nCRT in our patient cohort (Table 3).

Finally, we investigated potential changes in the miR-199b expression profile analyzing paired liquid biopsies from our initial cohort obtained after the neoadjuvant treatment. We could include post-treatment samples from 16 out of the 22 cases with enough material available. Interestingly, we found no differences in miR-199b expression levels between the subgroups of pre- and post-treatment samples (*p* = 0.649) (data not shown). Moreover, we observed decreased miR-199b levels with a fold change of 7.20 in post-treatment samples when we compared responders vs. non-responders, which is concordant with the results obtained in the pre-treatment specimens. Differences were statistically significant between subgroups (*p* = 0.038) (Figure S6).

## 3. Discussion

In recent years, many studies in the literature have evaluated the usefulness of miRs as liquid biopsy biomarkers. MiRs function as key regulators of diverse biological processes with high relevance in cancer, acting as oncogenes or tumor suppressors depending on their target genes, and have been involved in the pathogenesis of many tumor types [47,48,49,50]. Great insights into the implication of miRs in tumorigenesis and metastasis have allowed us to better understand the molecular mechanisms of disease development and progression, their potential clinical impact as biomarkers, and the investigation of miR-based therapies.

It has been reported that miR-199b is downregulated in several cancers and exerts tumor suppressive functions in tumor cell growth, invasion and metastasis [35,36,49,50]. In previous works from our group we have already studied the association of miR-199b with CRC progression, but it is still necessary to fully evaluate the biological role of miR-199b deregulation and its oncogenic properties. In the present work, we observed that miR-199b significantly reduces cell migration (Figure 1) and colony-forming ability (Figure 2). These results were in line with our initial hypothesis since miR-199b has been identified to negatively modulate SET [39], a key regulator of cell migration in CRC [51,52]. In fact, the potential role of the miR-199b/SET signaling axis in cell migration was also evaluated by transwell migration assays, observing that SET overexpression reversed the migration inhibitory effects of miR-199b (Figure S2). In fact, we show here that the ectopic expression of SET totally restores the miR-199b induced effects in colony formation ability (Figure S3), and the relevance of the miR-199b/SET axis was also evaluated and confirmed in cell proliferation (Figure S4). Furthermore, the observed effects of this miR in cell migration could also be explained by the fact that miR-199b also regulates additional targets such as JAG1 [31,32,38], DDR1 [35,36,38] or SIRT1 [42], which have been reported to be involved in the regulation of cell migration and invasion in CRC, as well as in other tumor types. Moreover, we analyzed the effect of miR-199b modulation on caspase-dependent apoptosis and we found a markedly enhanced number of apoptotic CRC cells after miR-199b overexpression. Thus, our results in Figure 3 are in concordance with previous observations reporting changes in CRC cell viability after ectopic miR-199b modulation [39]. These miR-199b-derived antitumor effects could probably be explained by its role as a negative regulator of several of those targets indicated above. We also showed that colonosphere formation ability was altered after miR-199b overexpression, substantially decreasing not only the number of colonosphere-derived cells formed (Figure 4), but also the number of cells per colonosphere (Figure S5). These observations could be due to the effect of miR-199b regulating targets such as SET or CD133 that have been described to regulate CRC cell stemness [34,37]. In fact, colonosphere derived CRC cells have been found to be enriched in CD133 expression, and to show a concomitant miR-199b downregulation. Of note, it can be noticed that in vitro experiments using anti-miR-199b showed that it had a significantly lower effect than pre-miR-199b. This issue can be explained by the fact that CRC cell lines including both SW480 and HT-29 have been described to express very low basal levels of this miR [39]. In fact, in concordance with the in vitro results showed in the present work, it has been previously reported that the same slight effects in both proliferation and modulating of PP2A activity exist with the anti-miR-199b due to this issue [39,44]. Notably, the miR-199b basal expression is even lower in SW480 cells than in HT-29 cells (as we also confirmed here, Table S1), which could explain that the effects of miR-199b silencing in cell migration that we found here only achieved significance in the HT-29 cell line (Figure 1). Altogether, the functional relevance of this miR is probably due to the contribution of several regulated targets of different signaling pathways, as explained above.

Regarding the clinical impact of miR-199b in LARC, we have previously studied its relevance as a prognostic tissue marker in LARC [44], results that were recently validated in a larger and independent cohort [45]. Moreover, miR-199b was also identified by Baek et al. [46] as one of the miRs with a potential clinical value as circulating preoperative marker in serum and exosomes-derived samples, determining its overexpression in higher survival rates. In concordance with the previous data and our findings with miR-199b as a tumor tissue marker in LARC patients, we found miR-199b downregulated in 22.7% of cases, compared with the prevalence of 26.4% and 22.2% obtained in our recent works [44,45]. Interestingly, we described that miR-199b downregulation associates with higher tumor size and higher pathological stage after nCRT (Table 1), as previously described [44]. We next stratified our cohort by the response to nCRT and observed lower miR-199b levels in the subgroup of non-responder cases (Figure 5). Notably, we also found that plasma expression levels of this miR had a predictive value of response to nCRT (Table 2), results that are similar to those obtained for the same miR as tumor tissue markers [44,45], and significantly associated with patient downstaging (Table 3). Moreover, we analyzed paired liquid biopsies after nCRT and also observed a marked reduction of miR-199b expression in the subgroup of non-responders (Figure S6), further supporting the findings described in pre-treatment samples. However, our findings here need to be confirmed in larger independent cohorts since the low number of cases analyzed in this work together with the lack of a training and a validation set of cases represents a strong limitation of the study, and the conclusions should be taken with caution.

The involvement of miR-199b deregulation in CRC progression has been previously reported in the literature. Thus, it has been described that miR-199b is downregulated in hepatic metastasis tissues from CRC patients [42], which was also described by our group in metastatic CRC [39] and LARC patients [45], observing that the subgroup of patients with low miR-199b expression showed markedly higher recurrence rates. These observations prompted us to explore the potential involvement of this miR in disease progression in liquid biopsies as well. We found a strong association between low miR-199b levels and relapse (Table S4), even though we did not reach statistical significance mainly due to the short cohort of patients that we could include in this study. Altogether, these findings strengthen the fact that miR-199b is hardly involved in tumor malignant features and progression, and could be used as a predictive circulating marker of response to nCRT.

Of note, there are some limitations of our study that have to be taken into consideration. These limitations include the need to validate the functional role of miR-199b observed using in vivo models and primary cultures from CRC patients. Moreover, the limited number of patients included in our study supposes a great limitation, and a future validation of these results in a larger, multicentric and independent cohort of LARC patients that would reinforce the clinical relevance of miR-199b as a circulating marker of response to nCRT in this disease is required. In addition, it would be interesting to lengthen the time of the study to analyze miR-199b as a predictor of patient outcome and relapse. This also could allow for the obtaining of periodic blood samples of each patient, thereby making an exhaustive follow-up of both responders and non-responders to nCRT groups. This would increase the number of patients that could avoid postoperative complications due to TME surgery and could be included in a W&W protocol, improving the quality of life of a larger number of selected patients. Moreover, an independent study with a larger W&W patient cohort could represent an opportunity to validate miR-199b as a circulating marker of recurrence in patients following this approach.

## 4. Materials and Methods

### 4.1. Cell Cultures and Transfection

The human CRC cell lines SW480 (ATCC CCL-228) and HT-29 (ATCC HTB-38) were purchased from American Type Culture Collection (ATCC, Manassas, VA, USA). Authentication was done by the authors in all cases (LGC Standards, Wesel, Germany). Cell lines were maintained in RPMI-1640 (Invitrogen, Carlsbad, CA, USA) with 10% fetal bovine serum (FBS) and were grown at 37 °C in a 5% CO_2_ atmosphere. Media were supplemented with penicillin G (100 U/mL) and streptomycin (0.1 mg/mL). For transfection experiments, CRC cells were seeded in 6-well plates and transfected with 10 µL of Lipofectamine 2000 (Invitrogen, Carlsbad, CA, USA) and 20 nM of a miR-199b specific mirVana™ miRNA Mimic and Inhibitor (Ambion, Cambridge, UK).

### 4.2. Patient Samples

The study was carried out on plasma samples obtained from a total of 22 LARC patients who were selected retrospectively and treated between 2018 and 2021 in University Hospital Fundación Jiménez Díaz (Madrid, Spain). Plasma samples were taken from each patient before and after preoperative nCRT. Baseline, clinical and pathological characteristics of each patient at the time of inclusion are presented in Table S3. All patients were treated with nCRT and TME, except for two of them who were not subjected to surgery and followed a therapeutic strategy based on the W&W protocol. All of them were treated by the European Guidelines Recommendations with correct preoperative locoregional staging based on a magnetic resonance (MR), a transrectal ultrasound (TRUS), and a full body computed tomography (CT). The selection criteria included the presence of adenocarcinoma with operable disease, enough material collected before and after nCRT, clinical follow-up data available and absence of metastasis. TNM (tumor, node, metastases) staging was performed based on the 7th American Joint Committee on Cancer (AJCC) staging system established for CRC. Plasma samples were collected into EDTA Vacutainer^®^-tubes and immediately sent to the laboratory. Samples were subjected to centrifugation at 1500× *g* for 15 min at 4 °C for plasma separation. The supernatant was carefully transferred to a sterile tube and centrifuged again at 3000× *g* for 5 min at 4 °C in order to pellet any debris and insoluble components. Plasma samples were aliquoted and immediately frozen at −80 °C until the experiments were carried out. All patients gave written informed consent for sample storage and analysis at Fundación Jiménez Díaz Hospital biobank with the approval of the ethical committee and institutional review board of Fundación Jiménez Díaz (ref. PIC202-20).

### 4.3. Evaluation of Pathological Response

All tumor samples that resulted from the initial biopsies derived from colonoscopy and the surgical resection were classified according to the College of American Pathologist guidelines for invasive carcinomas (TNM, 7th ed.). Two independent pathologists who were blinded to patient outcome evaluated tumor regression grade according to the modified Ryan classification that categorizes tumors into four levels of response: complete response, moderate response, minimal response and poor response. Complete response indicates no viable cancer cells (RYAN 0), moderate response indicates single cells or little groups of cancer cells (RYAN 1), minimal response denotes residual cancer outgrown by fibrosis (RYAN 2), and poor response is associated with minimal or no tumor kill with extensive residual cancer (RYAN 3). According to clinical guidelines, every regression grade was compared with the primary tumor [53].

### 4.4. Wound-Healing Assay

A total of 8 × 10^5^ cells per well were seeded in 6-well plates and allowed to adhere for 24 h in complete medium. The monolayer was artificially injured by scratching across the plate with a 10 µL pipette tip. Wells were then washed twice with phosphate-buffered saline (PBS) to remove detached cells and wound healing was monitored using a Leica DMi1 (Leica, Wetzlar, Germany) microscope and the image acquisition software Leica Application Suite version 4.5. Images were captured at the beginning and at regular intervals during cell migration to close the wound. Comparisons were performed to quantify the migration rate of the cells between the different experimental conditions. Relative cell migration is represented in the histograms considering the percentage of healed area after ectopic miR-199b silencing or overexpression and compared to control conditions.

### 4.5. Transwell Migration Assay

Migration assays were performed using 24-well plates with transwell permeable supports of 6.5 mm insert and a polycarbonate membrane with an 8 µm pore size (Costar 3422, Corning Inc., Corning, NY, USA). Cells were seeded in the upper chamber at 1 × 10^4^ cells/mL in 0.1 mL of serum-free RPMI-1640 media. A volume of 0.8 mL of media supplemented with 10% FBS was placed in the bottom well as a chemo-attractant. After incubation for 24 h at 37 °C in an atmosphere containing 5% CO_2_, migrated cells on the lower surface were stained using crystal violet and counted under a light microscope.

### 4.6. Colony-Forming Assay

Experiments were performed in 6-well plates coated with 3 mL of 0.6% soft agarose (Sigma, St. Louis, MO, USA) in RPMI medium. A total of 5 × 10^3^ cells were suspended in 0.3% agarose in RPMI medium and plated in triplicates over the pre-coated wells. Fresh medium was supplied twice a week. After 10–15 days, colonies were stained with Thiazolyl Blue Tetrazolium Bromiden MTT (M-5655, Sigma, St. Louis, MO, USA) for 4 h at 37 °C. Then, colonies were fixed by adding dimethyl sulfoxide (DMSO) overnight at 37 °C. Colony numbers were determined from triplicates and three independent experiments were carried out for each condition and cell line.

### 4.7. Cell Viability Assay

Cell proliferation was measured in triplicate wells by the MTS assay in 96-well plates using the CellTiter 96 Aqueous One Solution Cell Proliferation Assay (Promega Corp., Madison, WI, USA), according to the manufacturer’s instructions.

### 4.8. Analysis of Caspase Activation

Quantification of caspase-3/7 activity was carried out using the caspase Glo-3/7 assay kit (Promega Corp., Madison, WI, USA). First, 5 × 10^3^ cells were seeded in a white-walled 96-well plate, and the Z-DEVD reagent, the luminogenic caspase-3/7 substrate containing a tetrapeptide Asp–Glu–Val–Asp, was added with a 1:1 ratio of reagent to sample. After incubation at room temperature for 90 min, the substrate cleavage by activated caspase-3 and -7 and the intensity of a luminescent signal was measured by a FLUOstar OPTIMA luminometer (BMG Labtech, Cary, NC, USA). Differences in caspase-3/7 activity are expressed as fold-change in luminescence.

### 4.9. Colonosphere Formation Assay

We generated colonosphere-derived cells from SW480 and HT-29 using 6-well ultra-low attachment plates (Corning Inc., Corning, NY, USA), where 10,000 cells per well were plated. Cells were grown in serum-free medium DMEM/F12 supplemented with GlutMAX^TM^-I (ThermoFisher Scientific, Waltham, MA, USA), 1% N2 (ThermoFisher Scientific), 2% B27 (ThermoFisher Scientific), 20 ng/mL human FGF (Sigma, St. Louis, MO, USA) and 50 ng/mL EGF (Sigma). After seven days, plates were analyzed for colonosphere formation. For quantification of the number of cells per colonosphere, colonospheres were collected and dissociated with trypsin to give single-cell suspensions. Viable cells were counted in a Neubauer chamber using a Trypan Blue exclusion test.

### 4.10. Western Blot Analysis

Protein extracts were isolated using TRIzol Reagent (Invitrogen, Carlsbad, CA, USA) following manufacturer’s indications, clarified (12,000× *g*, 15 min, 4 °C), denatured and subjected to SDS-PAGE and Western-blot. Antibodies used were goat polyclonal anti-SET (E-15) (Santa Cruz Biotechnology, Dallas, TX, USA), and mouse monoclonal anti-βactin (Sigma, St. Louis, MO, USA). Proteins were detected with the appropriate secondary antibodies conjugated to alkaline phosphatase (Sigma, St. Louis, MO, USA) by chemiluminescence using Tropix CSPD and Tropix Nitro Block II (Applied Biosystems, Foster City, CA, USA).

### 4.11. RNA Isolation

RNA extraction from all plasma samples was performed using the Qiagen miRNeasy Serum/Plasma Kit (QIAGEN, Hilden, Germany, catalog number: 217184). Plasma was thawed on ice and centrifuged at 3000 g for 5 min at 4 °C. An aliquot of 200 μL of plasma per sample was transferred to a new microcentrifuge tube and 1 mL of Qiazol was added. Total RNA, including small RNAs, were enriched and purified following the manufacturer’s instructions. RNA obtained was quantified with a Nanodrop Spectrophotometer (Thermo Scientific, Waltham, MA, USA).

### 4.12. Quantification of miR Expression Levels

The RNA samples were reverse transcribed using the TaqManHMicroRNA Reverse Transcription Kit (Applied Biosystems, Foster City, CA, USA), and mature miRs were quantified by quantitative real-time reverse transcription polymerase chain reaction (RT-PCR) using TaqMan MicroRNA Assays (Applied Biosystems) specific for the miR-199b (reference number: 000500), and miR-1228 (reference number: 002919), which was used as internal control. Reactions were carried out using an Applied Biosystems 7500 Sequence Detection System under the following conditions: 95 °C for 10 min, followed by 40 cycles of 95 °C for 15 s and 60 °C for 1 min. Analysis of relative gene expression data was performed using the ΔΔCT method [54], where ΔΔCT = (CT, Target Gene-CT, control) Tumor-(CT, Target Gene-CT, control) Normal Control. MiR-199b downregulation was considered when the expression in a sample was lower than the mean minus standard deviation (SD) of the patient cohort, as previously described [39].

### 4.13. Statistical Analysis

All statistical analyses were carried out using the software tool SPSS v20 for Windows (SPSS Inc., Chicago, IL, USA). The association between miR-199b expression and clinical and molecular parameters were analyzed by applying the chi-square test (Fisher’s exact test) based on bimodal distribution of data. Comparisons between miR-199b expression levels of each patient before and after preoperative CRT were performed using Mann–Whitney and paired t-tests. Data represented for transfection experiments are mean of three independent experiments ± SD. Statistical comparisons were obtained by two-sided t-test analyses. Statistical significance was considered when p-value was lower than 0.05. This study has been performed following the Reporting Recommendations for Tumor Marker Prognostic Studies (REMARK) guidelines [55,56].

## 5. Conclusions

In conclusion, our findings here provide novel important data regarding the relevance of miR-199b as a regulator of cellular processes crucial for malignant transformation such as cell growth, migration, aggressiveness, colonosphere formation and apoptosis, with the particular importance of the miR-199bSET axis in some of these phenotypes. At the clinical level, we found that miR-199b expression could serve as a marker in plasma samples from LARC patients, showing predictive value of response to nCRT and patient recurrence. Altogether, our results highlight the functional roles and clinical usefulness of miR-199b as a potential circulating biomarker in LARC patients.

## Figures and Tables

**Table 1:** Association of miR-199b expression with clinical and molecular characteristics in a cohort of 22 liquid biopsies from LARC patients.

Parameter	No. Cases	No. miR-199b High (%)	No. miR-199 Low (%)	*p*
MiR-199b	22	17 (77.3)	5 (22.7)	
Gender	22	17	5	0.078
Male	12	11 (91.7)	1 (8.3)	
Female	10	6 (60)	4 (40)	
Age	22	17	5	0.962
<70	13	10 (76.9)	3 (23.1)	
≥70	9	7 (77.8)	2 (22.2)	
Grade pre-CRT ^1^	22	17	5	0.211
Low	14	12 (85.7)	2 (14.3)	
Moderate-High	8	5 (62.5)	3 (37.5)	
Clinical stage pre-CRT	22	17	5	0.312
II	3	3 (100)	0 (0)	
III	19	14 (73.7)	5 (26.3)	
ECOG ^2^	22	17	5	0.962
0	13	10 (76.9)	3 (23.1)	
1	9	7 (77.8)	2 (22.2)	
ypT ^3^	20	15	5	0.002
0–2	12	12 (100)	0 (0)	
3–4	8	3 (37.5)	5 (62.5)	
ypN ^4^	20	15	5	0.091
0	14	12 (85.7)	2 (14.3)	
1–2	6	3 (50)	3 (50)	
Pathological stage	20	15	5	0.020
yp0-I	9	19 (100)	0 (0)	
ypII-III	11	6 (54.5)	5 (45.5)	

^1^ CRT = Chemoradiotherapy; ^2^ ECOG = Eastern Cooperative Oncology Group; ^3^ ypT = tumor size after CRT; ^4^ ypN = pathological lymph node after CRT.

**Table 2:** Association between response to nCRT and miR-199b expression in liquid biopsies from LARC patients.

Response to Neoadjuvant CRT ^1^				
Response	No. Cases	Responders ^2^ (%)	Non-Responders ^3^ (%)	*p*
MiR-199b expression	22	11	11	0.011
Low	5	0 (0)	5 (100)	
High	17	11 (64.7)	6 (35.3)	

^1^ CRT = chemoradiotherapy; ^2^ Responders = cases with moderate or complete pathological response, and cases following W&W strategy; ^3^ Non-Responders = cases with poor or minimal pathological response.

**Table 3:** Association between downstaging and miR-199b expression in liquid biopsies from LARC patients.

Downstaging	No. Cases	Yes (%)	No (%)	*p*
MiR-199b expression	22	15	7	0.009
Low	5	1 (20)	4 (80)	
High	17	14 (82.4)	3 (17.6)	

## Data Availability

Data sharing is not applicable for this article.

## Supplementary Materials

The following are available online at https://www.mdpi.com/article/10.3390/ijms23042203/s1.
