# Supplementary material for: MicroRNA-199b Deregulation Shows Oncogenic Properties and Promising Clinical Value as Circulating Marker in Locally Advanced Rectal Cancer Patients
Source: Int J Mol Sci. 2022 Feb 17;23(4):2203. doi: 10.3390/ijms23042203 (PMC8875596; doi:10.3390/ijms23042203)
Supplement: Supplementary file 1 [file ijms-23-02203-s001.zip › ijms-1554580-supplementary.pdf]

MicroRNA-199b deregulation shows oncogenic properties and promising clinical value as circulating marker in locally advanced rectal cancer patients

Andrea Santos [1,2,†], Ion Cristóbal [1,2,*,†], Jaime Rubio [1,2,3,†], Cristina Caramés [1,2,3], Melani Luque [4], Marta Sanz-Alvarez [4], Miriam Morales-Gallego [4], Juan Madoz-Gúrpide [4], Federico Rojo [4] and Jesús García-Foncillas [2,3,*]

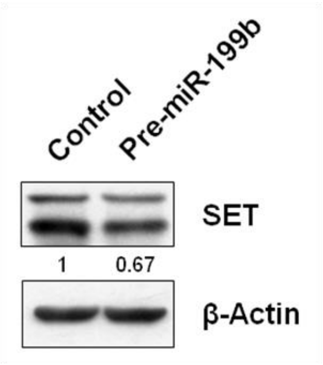

Figure S1. Western blot analysis confirming the role of miR-199b as a negative regulator of SET in SW480 cells ectopically expressing the pre-miR-199b.

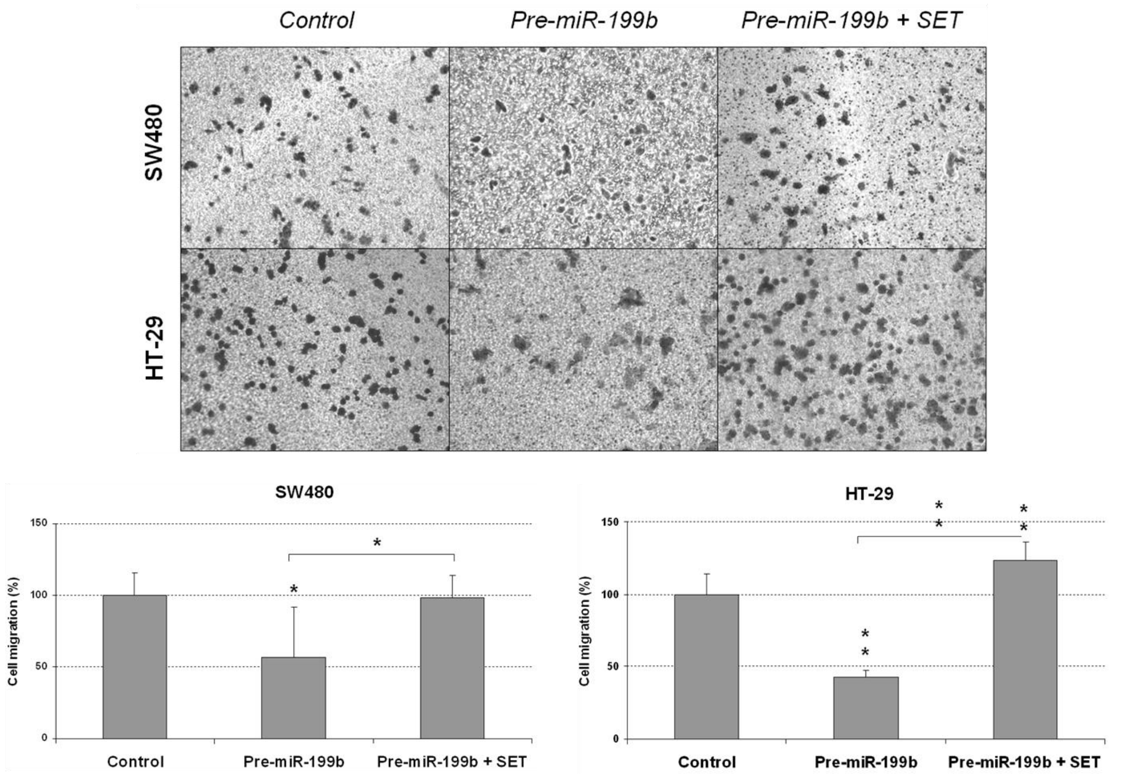

Figure S2. Transwell migration assay in miR-199b-expressing SW480 and HT-29 cells with or without SET overexpression; * $p < 0.05$; ** $p < 0.01$.

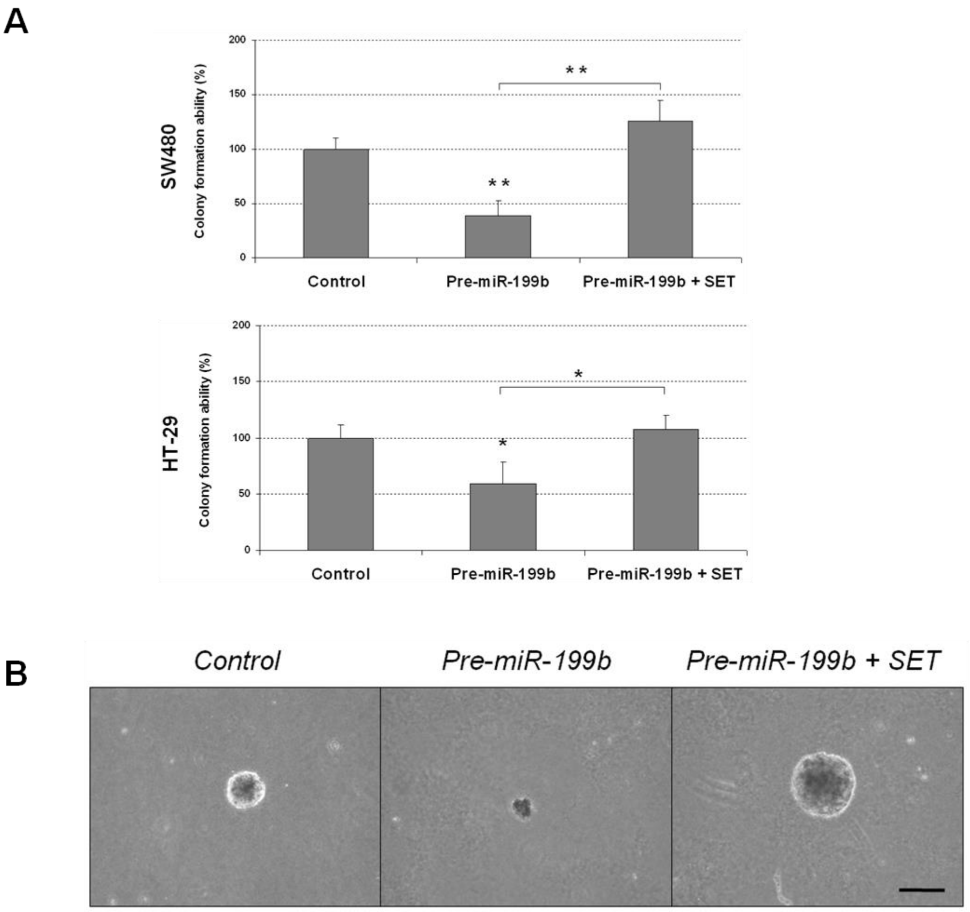

Figure S3. Role of the miR-199b/SET signaling axis regulating colony-forming ability of CRC cells. (A) Colony-forming assays showing the effect on the anchorage-independent cell growth of miR-199b-expressing SW480 and HT-29 cells with and without SET overexpression; (B) Optical microscope images showing differences in the size of the SW480-derived colonies; * $p < 0.05$; ** $p < 0.01$. Scale bar: 100 μm.

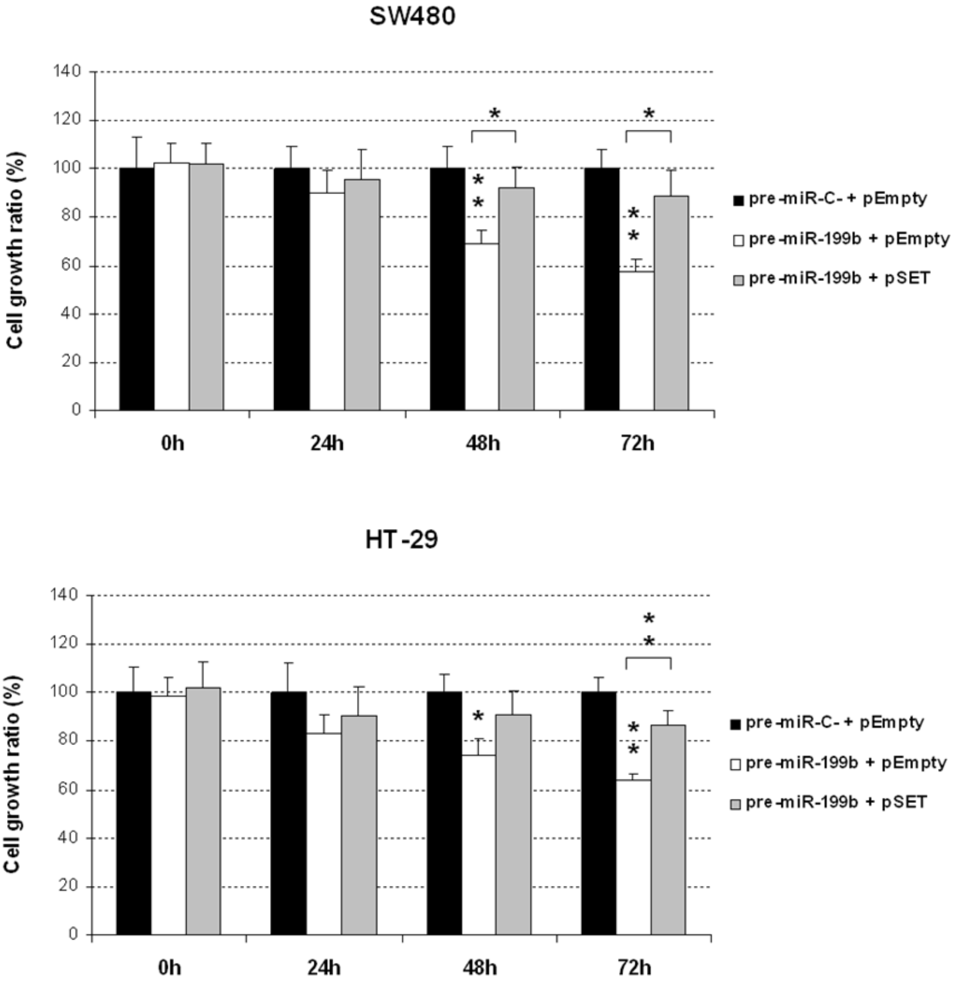

Figure S4. MTS assay showing the role of the miR-199b/SET signaling axis regulating CRC cell proliferation; * $p < 0.05$; ** $p < 0.01$.

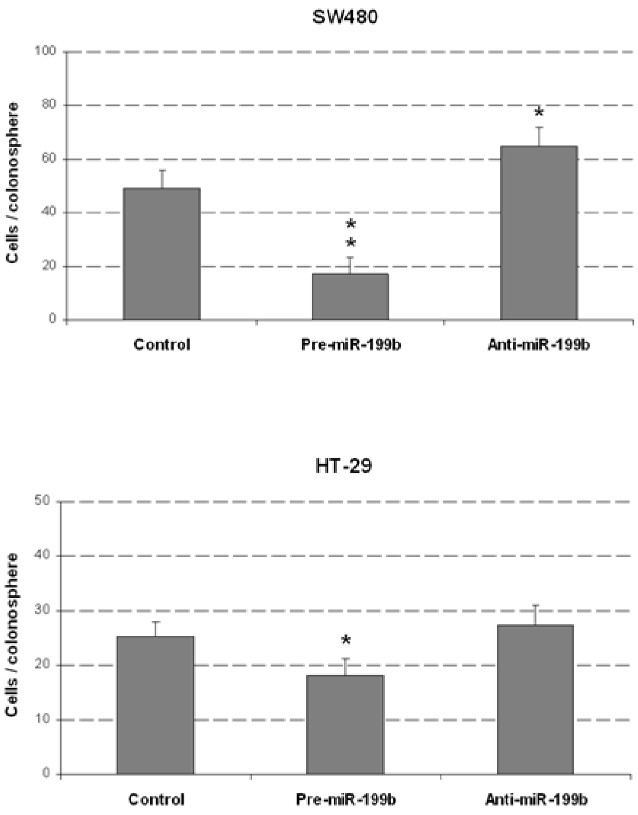

Figure S5. MiR-199b reduces the number of cells per colonosphere formed in CRC cells. The graphs show number of cells per SW480 and HT-29-derived colonospheres obtained after transfection with pre-miR-199b and anti-miR-199b; * $p < 0.05$; ** $p < 0.01$.

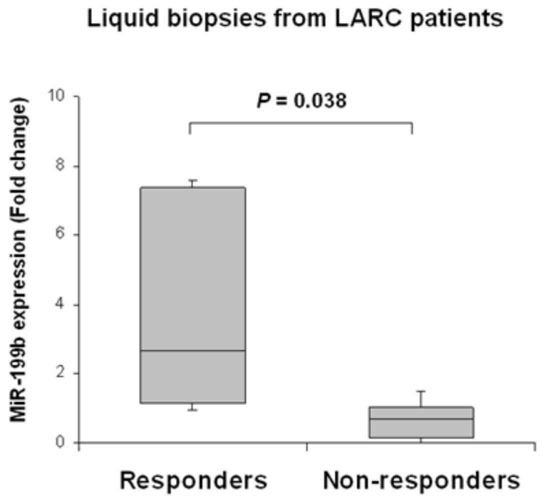

Figure S6. MiR-199b low expression levels associates with lack of response to nCRT in post-treatment samples stratified by the grade of response to nCRT. Responder group corresponds to cases with moderate or complete response (RYAN 0 and 1), including cases following W&W protocol. Non-responder group includes those with minimal or none complete response (RYAN 2 and 3).

Table S1. Quantification of miR-199b expression levels in SW80 and HT-29 cells.

$\Delta\Delta C_T = (C_{T, miR\text{-}199b} - C_{T, miR\text{-}1228})_{Pre/Anti\text{-}miR\text{-}199b} - (C_{T, miR\text{-}199b} - C_{T, miR\text{-}1228})_{Control}$.

Cell line	Pre-miR-199b (−$\Delta\Delta C_T$)	Anti-miR-199b (−$\Delta\Delta C_T$)
SW480	11,73	-1,40
HT-29	10,69	-2,62

Table S2. Clinical and molecular characteristics of a serie of 22 LARC patients with liquid biopsies available.

Parameter	No. (%)	
Sex		
Male	12	(54.5)
Female	10	(45.5)
Age		
<70	13	(59.1)
≥70	9	(40.9)
ECOG[1]		
0	13	(59.1)
1	9	(40.9)
Site of primary tumor		
Rectum	22	(100)
Clinical stage pre-CRT[2]		
II	3	(15)
III	19	(85)
Grade pre-CRT		
Low	14	(63.6)
Moderate-High	8	(36.4)
ypT[3]		
0-2	12	(60)
3-4	8	(40)
ypN[4]		
0	14	(70)
1-2	6	(30)
Pathological stage		
yp0-I	9	(45)
ypII-III	11	(55)

[1]ECOG= Eastern Cooperative Oncology Group; [2]CRT= Chemoradiotherapy; [3]ypT= tumor size after CRT; [4]ypN= pathological lymph node after CRT.

Table S3. Clinical and molecular characteristics of each LARC patient included in the study.

Case	Sex	Age	ECOG[1]	Grade pre-CRT[2]	Clinical stage pre-CRT	ypT[3]	ypN[4]	Pathological stage
P1	F	80	1	G2	III	T3	N1	III
P2	M	50	0	G1	III	T0	N1	III
P3	F	77	1	G2	III	T2	N0	I
P4	M	61	0	G2	III	T2	N0	I
P5	M	49	0	G1	III	T2	N0	I
P6	F	55	0	G1	III	T2	N0	I
P7	M	65	0	G1	III	T0	N0	0
P8	M	60	1	G2	III	T2	N0	I
P9	F	68	1	G1	III	T2	N1	III
P10	M	73	1	G1	II	T2	N0	I
P11	F	61	1	G1	III	T2	N0	I
P12	M	71	1	G1	III	T2	N0	I
P13	M	76	0	G1	III	W&W[5]	W&W	W&W
P14	F	66	0	G1	III	T4	N0	II
P15	F	58	1	G2	III	T4	N2	III
P16	M	53	0	G1	II	T3	N1	III
P17	M	79	0	G2	III	T0	N0	0
P18	F	72	0	G1	II	T3	N0	II
P19	M	66	0	G2	III	T	N1	III
P20	M	77	0	G2	III	W&W	W&W	W&W
P21	F	72	0	G1	III	T3	N0	II
P22	F	65	1	G1	III	T3	N0	II

Table S3. Clinical and molecular characteristics of each LARC patient included in the study.

[1]ECOG= Eastern Cooperative Oncology Group; [2]CRT= Chemoradiotherapy; [3]ypT= tumor size after CRT; [4]ypN= pathological lymph node after CRT; [5]W&W: patients who follow a therapeutic strategy based on "watch and wait" protocol; M: male; F: female.

Table S4. Association between patient relapse and miR-199b expression in liquid biopsies from LARC patients.

Recurrence	No. cases	Yes	(%)	No	(%)	*p*
MiR-199b expression	22	3		19		0.051
Low	5	2	(40)	3	(60)	
High	17	1	(5.9)	16	(94.1)	
